# TMFoldWeb: a web server for predicting transmembrane protein fold class

**DOI:** 10.1186/s13062-015-0082-5

**Published:** 2015-09-17

**Authors:** Dániel Kozma, Gábor E. Tusnády

**Affiliations:** Institute of Enzymology, Research Centre for Natural Sciences, Hungarian Academy of Sciences, PO Box 7, H-1518 Budapest, Hungary

**Keywords:** Transmembrane protein, Fold recognition, Statistical potential, Ready for large-scale analysis

## Abstract

**Background:**

Here we present TMFoldWeb, the web server implementation of TMFoldRec, a transmembrane protein fold recognition algorithm. TMFoldRec uses statistical potentials and utilizes topology filtering and a gapless threading algorithm. It ranks template structures and selects the most likely candidates and estimates the reliability of the obtained lowest energy model. The statistical potential was developed in a maximum likelihood framework on a representative set of the PDBTM database. According to the benchmark test the performance of TMFoldRec is about 77 % in correctly predicting fold class for a given transmembrane protein sequence.

**Results:**

An intuitive web interface has been developed for the recently published TMFoldRec algorithm. The query sequence goes through a pipeline of topology prediction and a systematic sequence to structure alignment (threading). Resulting templates are ordered by energy and reliability values and are colored according to their significance level. Besides the graphical interface, a programmatic access is available as well, via a direct interface for developers or for submitting genome-wide data sets.

**Conclusions:**

The TMFoldWeb web server is unique and currently the only web server that is able to predict the fold class of transmembrane proteins while assigning reliability scores for the prediction. This method is prepared for genome-wide analysis with its easy-to-use interface, informative result page and programmatic access.

Considering the info-communication evolution in the last few years, the developed web server, as well as the molecule viewer, is responsive and fully compatible with the prevalent tablets and mobile devices.

**Reviewers:**

This article was reviewed by Dr. Michael Gromiha, Dr. Sandor Pongor and Dr. Frank Eisenhaber with Wing-Cheong Wong.

## Implementation

### Background

According to the sequence-structure-function paradigm, in order to get insight and fully understand the biochemical processes of the living cells, identifying currently unknown protein structures is indispensable. Since the Anfinsen experiment established that all the necessary information for structure formation is encoded in the protein sequence; scientists keep looking for an ultimate description and developing tools for structure prediction with increasing accuracy. However, a complete theoretical model is a longstanding goal of molecular biologists and biophysicists; due to the complexity of theoretical structure prediction, a universal solution has not been developed yet.

Currently, numerous protein structure prediction web servers with different approaches are available over the internet, but only a small part of them can be applied to transmembrane proteins (TMPs). For example, threading-based GenTHREADER and pGenTHREADER [1, 2] are widely used as accurate structure predictors, but they mask transmembrane regions, making the generated results uncertain for membrane regions. Another threading method, used for globular proteins is the RaptorX suite, which has an insufficient precision on TMPs, according to our previous benchmark [3].

Sequence-based approaches, such as HHsuite [4, 5], have a remarkable performance both in speed and accuracy, but theirs inherent limitation by a critical sequence identity cannot be eliminated, even if this threshold is low for an advanced method.

Currently there is not enough computational resource to carry out comprehensive modeling of protein structures with de novo methods, particularly for large proteins like TMPs. CABS-fold [6] and QUARK [7] allow query sequences only shorter, than 120 and 200 residues, respectively. According to the Human Transmembrane Proteome (HTP) database [8], the average chain length of TMPs in the human proteome is ~550 residues. Therefore, these two *de novo* methods are usable only for the 3.5 and 11 % of the sequences, respectively, let alone that their results are not always correct.

Here we present a fast and reliable fold predictor web server, TMFoldWeb, which is free from the need of the exhaustive conformation space mapping and the limitation of sequence identity.

### Algorithm

The predictor engine of the TMFoldWeb web server was described in our recent paper [3]. Here we briefly summarize the main properties and steps of the TMFoldRec algorithm. The main steps of TMFoldRec are the following: for a query sequence, a multiple sequence alignment is generated by PSI-BLAST [9]. In addition to the sequence of TMPs, it uses the predicted or the user provided topologies of TMPs as input and a statistical potential to describe the energy contribution of amino acid - amino acid and amino acid - lipid contacts. The topology information can be predicted using the built-in CCTOP method [8, 10]. The 20 standard amino acids are taken into account and the lipid membrane is handled as a homogeneous, continuous environment. Each interaction type was determined in a maximum likelihood procedure. TMFoldRec selects TMP structures with the same number of membrane regions from a previously collected representative structure database. Structures in this representative database contain the whole membrane-embedded quaternary structure of the TMPs. Therefore, TMFoldRec can take different oligomer forms and environments of TMPs into account to determine the most likely native fold. The knowledge of the surroundings has a considerable effect on the energy calculation and hence on the correct ranking of the various template structures, as well. For homo-oligomer structures the effect of the surrounding chains is taken into account with a periodic boundary condition corresponding to the symmetry of the biomolecule; at hetero-oligomer structures the neighboring chains are replaced by a previously determined, *z*-coordinate dependent average amino acid distribution.

The TM parts of the query sequence profile are aligned systematically onto the structures with same number of TM regions and the energy is calculated for each combination. The structure with the lowest energy is selected as the most probable fold candidate. In the 77 % of the cases native folds were ranked at the first position, i.e., they were assigned to the lowest energy over the alternative folds.

### User interface

TMFoldWeb has a simple and intuitive web interface. To submit a job, the user should enter the query sequence in FASTA format and the corresponding topology into the textboxes. Optionally, an automatic topology prediction can be performed using the newly developed CCTOP web server [8, 10].

On submission, the user is redirected to an information page, which monitors the submitted job in real-time and displays the current status and the finished percentages of the whole prediction process with a progress bar.

**Fig. 1:**
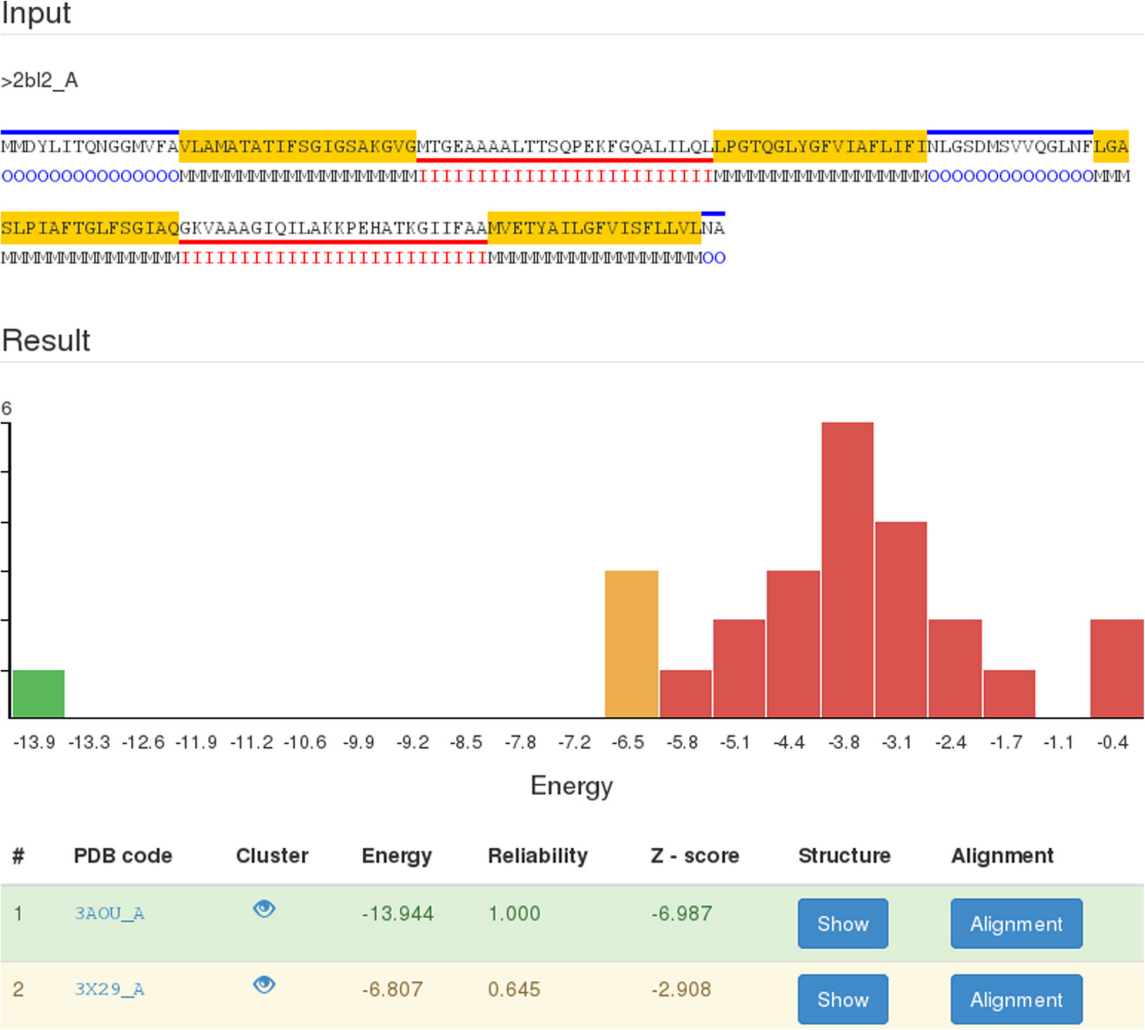
The output of the example query sequence. Elements from up to down: input sequence and its (predicted) topology (M: membrane, I: inside, O: outside), energy histogram, predicted templates ordered by energy values. The red and blue lines under and above the input sequence denotes the inside and outside localization of the given sequence parts, respectively. On the result panel the energy distribution of the templates is shown, and it is colored by the reliability of the hits: green: acceptable hits (reliability > 0.8), orange: possible hits (0.8 < reliability < 0.6), red uncertain hits (reliability < 0.6)

When the threading is finished, a result page (Fig. 1) appears summarizing all the information on the submitted sequence, including input sequence with the user provided or the predicted topology. Resulting fold candidates are sorted by the calculated energy and reliability values. The calculated energy values are summarized in a histogram to enable easy interpretation of the significance of a predicted native structure.

For each template the PDB ID and chain, fold class name, lowest energy value of the given template, reliability and the Z-score (the raw energy and the population mean energy in the units of the standard deviation) of the resulting energy values are presented. By clicking on the ‘Show’ button, the template chain with its protein and lipid environment can be displayed in the PV [11] molecule viewer. When the user clicks on the ‘Alignment’ button, the corresponding sequence-to-structure alignment appears in the drop-down row. The displayed alignment contains the whole query sequence, but only the membrane embedded regions of the structures.

Each row of the summarizing result table is colored corresponding to its reliability score. Templates with reliability greater than 0.8 are filled with green (certain results), values between 0.8 and 0.6 with yellow (possible good results) and rows bellow 0.6 are red (uncertain results).

Submitted job is stored for a week in the browser session to easily backtrack recent activity. This five-column-table is displayed at the bottom of the pages containing date of submission, name of the sequence, real-time status (if parallel processes are submitted) and the link to the result page. With the provided red icon, users can delete their unused/unwanted jobs.

**Fig. 2:**
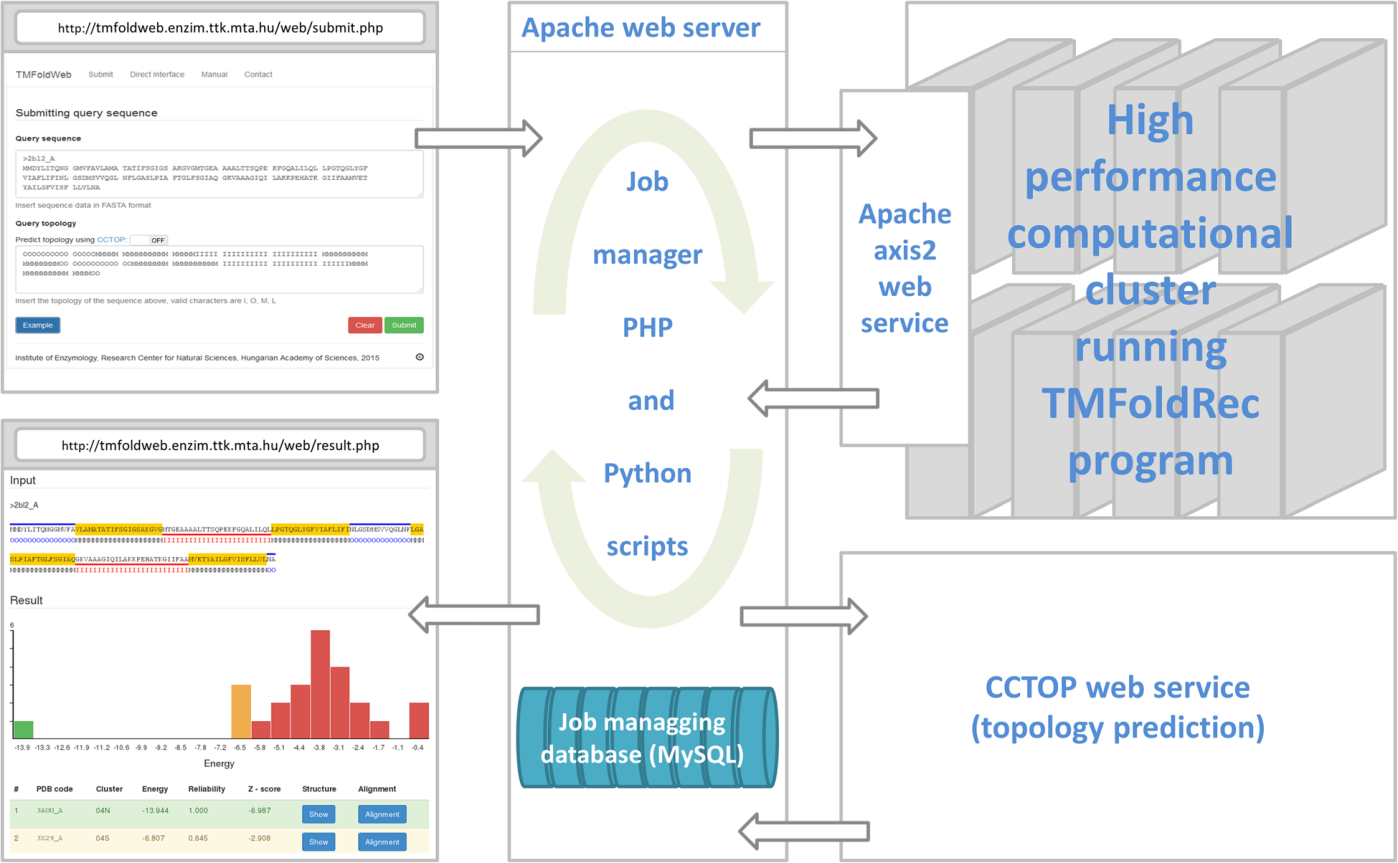
The schematic build-up of the webserver and its service. Here we summarize the workflow of the web server. User requests are inserted into a database storing jobs. A Python script periodically checks if new request is inserted. If the submission does not contains topology, sequence is submitted to the CCTOP web service. After completion, sequence and topology are delegated to the TMFoldRec program on our HPC cluster via WSDL, which returns a list of templates ordered by the energy and reliability values

The user interface of the web server is written in PHP, JavaScript with the Bootstrap CSS library and utilizes PV [11], a WebGL-based protein viewer. With a developed multilayer application the entered queries are inserted into a job management database. A job manager scripts written in Python, delegates submissions to a high performance cluster via an internal, private web service using WSDL (Fig. 2).

A programmatic access is also available via the direct interface for developers or for submitting genome-wide data sets.

### Comparison with HHsearch

To present the usability of TMFoldWeb we have compared it with HHsearch, the one of the most used remote homology detecting method. We have selected 2071 TMPs with more than two membrane regions and without structural evidence (cross reference to the PDB database) from the HTP database. We have built a custom database from the PDBTM database, according to the manual of HHsuite, to search remote structural homologues. The 2071 sequences were searched against the database as well as submitted into TMFoldWeb. We have tested if the best hit according to HHsuite has the same number of transmembrane segments than it were determined in the HTP database. In 987 out of 2071 cases, HHsearch predicted a model that has the same number of transmembrane regions as in the HTP database, but in the rest of the cases large differences could be observed, let alone the huge sequence length deviations. If we excluded hits with HHsearch probability below 80 %, we still get 740 incorrect predictions out of the 1690 cases. Moreover, there were 57 cases when HHsearch returned a hit with high probability, but TMFoldRec reports topological mismatch with all available templates.

These results highlight that while HHsuite takes into account the globular parts of the proteins as well, there are scenarios when this inclusion rather misleads the prediction at least from the point of view of TMPs. Since the target identification mainly based on the globular parts (in average ~75 % of the sequences is non-membrane regions) applying directly these hits could result in erroneous models for the structure of a transmembrane region.

### Discussion

Although the sequence similarity threshold for transmembrane segments of homologous TMPs could be much lower than in the case of globular proteins [12], the opposite of this statement has not been investigated so far. That is, what is the relation of the two TMPs if the sequence identity of the transmembrane segments is low. Perhaps this lower sequence identity threshold is the consequence of the lower sequence complexity of transmembrane regions. Therefore, other information is needed to establish the relationships between TMPs, for example considering the number of transmembrane segments in addition to the sequence identity, as it was shown in the previous section. To underline the necessity of a reliable fold prediction method, we have investigated the accuracy of HHsuite, one of the most accurate alignment method used for remote homology detection. We think, HHsuite is an outstanding program package for generating sequence alignments and searching databases for remote homologues, but it seems a little bit optimistic in the fold assignment of TMPs. Therefore, applying only TMFoldWeb in cases, when no sequence relative exists or using the reliability score of TMFoldRec besides the HHsearch probability can help to make more reliable fold prediction for TMPs.

## Availability and Requirements

- Project name: TMFoldWeb
- Project home page: tmfoldweb.enzim.ttk.mta.hu
- Operating system: platform independent
- Programming language: PHP, JavaScript, Python
- Other requirements: browser with WebGL support
- License: Attribution-NonCommercial 4.0 International
- Any restriction to use by non-academics: not allowed

## Reviewers’ comments

### Reviewer comment 1: Dr. Michael Gromiha

Summary: It is a good work about the development of a web server for predicting the folding of transmembrane proteins

Recommendations: In this work, the authors developed a web based tool, TMFoldWeb for predicting the topology of transmembrane proteins and sequence to structure alignment. The probable templates are listed at the output based on their energy values.

**R1:** The server takes a long time for displaying the output. The approximate time to be taken for obtaining the data may be displayed. It is not clear whether it depends on the size of the sequence and topology of the protein.

***Authors’ response:****The calculation time of a given query is depends on the number of membrane region exponentially and on the number of templates linearly. The estimated remaining time is hard to compute since these jobs run on a high performance compute grid besides many other projects.*

**R1:** The details for the abbreviation O, M and I could be given under the figure.

***Authors’ response:****Thank you for your advice, we have added the explanation to the figure caption.*

**R1:** The sequence contains blue, yellow and red lines. The energy profile contains green, yellow and red. Are there any mapping between the two?

***Authors’ response:****There is no connection between the two color schemes. But to avoid ambiguousness we have added an explanation to the figure caption.*

**R1:** Several PDB codes are given as probable templates. The most appropriate one may be highlighted (based on reliability?).

***Authors’ response:****On the result page of TMFoldWeb the results are ordered by the energy values. Since the reliability score is a monotonous function of the energy the first row (if we keep the number of transmembrane segments constant), containing the lowest energy hit is the most reliable prediction as well. The color scheme helps to find the most appropriate template as well, and we have added a description on the figure legend as well.*

**R1:** Are there any correspondences between alignment and energy?

***Authors’ response:****The energy values in each rows correspond to the best sequence-to-structure alignment achieved on the given template structure.*

Minor issues: mentioned above

### Reviewer comment 2: Dr. Sandor Pongor

Summary: The work is acceptable.

Recommendations: In this manuscript Kozma and Tusnady describe a new web server, which uses the TMFoldRec algorithm to recognize the fold of transmembrane proteins. The topic of membrane protein fold recognition is very important as currently most folds are yet unknown and it is estimating that it will take a couple more decades for a good coverage of membrane protein fold space. It was shown, that the accuracy of the presented method is very high (77 % of the cases the native folds were identified accurately), which accuracy is far the highest among the recent methods. Moreover, the reliability estimation of the lowest energy model is very important feature of the method from the viewpoint of whole genome structure prediction of transmembrane proteins. The description of the web server is convincing, the manuscript is well written and sufficient details have been provided. Since the authors applied the most novel programming techniques, it can be used even on my smartphone.

**Minor issues:** I suggest the following minor points for improvements:

**R2:** The progress bar was stuck in the beginning of the prediction process at 2 % and was moving only during the threading procedure.

***Authors’ response:****Thank you for your advice, we have integrated the status feedback of CCTOP topology predictor.*

**R2:** The direct interface is a great idea for programming access the web server, and works well, however the output is somewhat ugly, a more formatted output should be better (for example using print “Status: {}, percent: {}”.format(stat[‘message’],stat[‘percent’]) instead of the simple print function on line 70).

***Authors’ response:****Thank you for your advice, we have modified the corresponding script lines in our minimal working example.*

**R2:** On the result page the name of the clusters are indicated, but we do not know the elements of the clusters. It should be nice, if the user can resolve the content of the clusters by clicking to the cluster name.

***Authors’ response:****We have included this requested feature to the server as well.*

**R2:** Also a link to the appropriate PDB entry may help the navigation on the result page.

***Authors’ response:****Thank you, the link to PDBTM is added on the result page.*

**R2:** The description of Z-score is missing both from the manuscript and from the manual.

***Authors’ response:****We have added a short explanation into the manuscript as well as into the manual.*

**R2:** It is possible to insert topology with re-entrant loop on the submit page? If yes, how?

***Authors’ response:****Of course, our algorithm is capable to predict transmembrane proteins with re-entrant regions. It is automatically predicted, if the user chooses the CCTOP prediction, or can be entered as input topology by giving the same localization before and after the membrane region (e.g. …IIIIMMM…MMMIII… for intracellular re-entrant loop and …OOOMMM…MMMOOO… for extracellular re-entrant loop, respectively).*

### Reviewer comment 3: Dr. Frank Eisenhaber and Wong Wing-Cheong

Summary: This review was discussed and written by Wong Wing-Cheong and Frank Eisenhaber. Based on the authors’ recently published software work (TMFoldRec, BMC Bioinformatics (2015)16:201), Kozma et al. wrote an interface for their TMFoldRec software and presented it as a webserver, herein, TMFoldWeb (available at tmfoldweb.enzim.ttk.mta.hu). The webserver is designed specifically at the task of predicting the fold of α‑helical transmembrane (TM) proteins. Generally speaking, the web interface itself is well‑written and it could potentially be a useful resource for the scientific community if the following major issues are addressed.

**Major Issues:**

**R3:** Demonstration of TMFoldWeb’s utility: Currently, the demonstration of utility is through contrasting TMFoldWeb against HHsearch on the PDBTM database. However, the performance evaluation based on the number of predicted TM helices out of the total TM helices of the entry appears incomplete since.

(i) HHsearch operates on local alignment mode by default.

***Authors’ response:****We have tested HHsearch with both local and global search options, but the results using local alignments were a greater correspondence with the results of the highly reliable CCTOP predictions.*

**R3:** (ii) even if the number of TM helices are predicted correctly, it does not imply that the correct TM fold necessarily follows. In hindsight, since the fundamental concept of TMFoldRec has already been previously published, this derivative article should aim to present itself as an important utility (novelty and utility as the criteria of the application note section) by demonstrating examples of practical usage to the potential users. It would be especially insightful to provide some biological examples where other methods (e.g. HHsearch, RaptorX) had failed while TMFoldWeb was able to derive plausible fold for the query sequence.

***Authors’ response:****The comparison to HHsearch would aim to present the scientific significance of TMFoldWeb. In our previous paper we have benchmarked its core engine, TMFoldRec and it has significantly overcome other programs in fold prediction. The usability and importance of the method as well as the inherent danger of using a general method was intended to show through a blind prediction on the human transmembrane proteome. According to our view, this solution is a better representation of the usability of the TMFoldWeb server, than showing it is superior to other methods on one spectacular example.*

**R3:** Rigor of TMFoldWeb:

Though TMFoldWeb is aimed squarely at predicting the fold of the TM proteins, its efficiency at avoiding/minimizing fold predictions of α-helical globular proteins is unclear. To backtrack, TMFoldRec itself has shown to perform well against the more generalized (e.g. HHalign, Jackhammer, RaptorX, pGenTHREADER) methods on a set of 124 TM proteins (constrained to >2TM and <16TM derived from PDBTM database) as positive controls, hence its good sensitivity. However, on the flip side, TMFoldWeb’s false-positive rate (i.e., specificity) when presented with α-helical globular sequences (for example. SCOP α-helical protein class as negative controls) remains uncharacterized. Typically, a compromise between sensitivity and specificity has to be made with respect to any algorithm. A small set of case studies based on TMFoldWeb found that the globular sequences of a cytochrome c (pdb:1H21|A) and a nisin cyclase (pdb:2G0D|A) were predicted to have the TMfolds of an ATP synthase (pdb:4CBK|A; reliability = 1.000) and an ATPase (pdb:3AOU|A; reliability = 0.721) respectively (see 4 figures in extra file). In fact, false predictions with the reliability value of 1 can occur for sequences up to 50 % sequence identical to pdb:1H21|A (e.g. WP_029435684, 70 %ID; WP_005988336.1,60 % ID; WP_012176431.1,52 % ID; see 3 figures in extra file). On the other hand, the TM sequence of a ATPase subunit C (pdb: 1C17|M) did not find itself and its closest hit is a hydrogenase (pdb:4GD3|A; reliability = 0.586; see 1 figure in extra file). Indeed, both over- and under-prediction had occurred. As such, establishing error rates in terms of sensitivity/specificity associated with each observed reliability value seems reasonable for the users to make proper judgment of the predictions. To note, we have to assume that the users’ query sequence is uncharacterized and there is usually no alternative way to know about the real fold. As an additional point, the TM fold predictions of “cytochrome c/ATP synthase” and “nisin cyclase/ATPase” were concluded from the alignments of 2 α-helix/TM-helix and 4 α-helix/TM-helix matches respectively. In both cases, the aligned segments made up barely 12 and 20 % of the query sequences. Since the motivation of fold prediction is to infer the function (via the concept of sequence-fold-function) of the characterized protein to the query sequence, the transfer of function/fold on the basis of some local similarity, even if statistically significant, is not a strong argument for homology. For users who are unfamiliar with the sequence homology concept, the results can be misleading. The authors might look up the following publications for arguments why similarity within TM regions is a weak argument for homology: Biol Direct. 2015 Aug 1;10(1):39, BMC Bioinformatics. 2014 Jun 2;15:166, Nucleic Acids Res. 2012 Jul;40(Web Server issue):W370-5, Biol Direct. 2011 Oct 25;6:57, PLoS Comput Biol. 2010 Jul *29;6(7):e1000867.*

***Authors’ response:****Thank you for the detailed critics. Specificity and sensitivity are indeed important measures. Since TMFoldRec relies on the results of CCTOP topology predictors, we did not address the discrimination of transmembrane and globular protein in this manuscript. As it was shown previously, CCTOP have both value at 99 % (Biology Direct 2015, 10:31; Nucl. Acids Res. 2015, 43:W408-12). Therefore, these values are the same for TM filtering in TMFoldRec.*

*Regarding the issue of 1h21:A and 2g0d:A, these structures has two fully buried helix located in a hydrophobic environment of the protein, which might mislead the predictors. According to the result of CCTOP prediction all of the state-of-the-art topology prediction methods utilized in CCTOP failed on the sequence of 2g0d:A (see figure below).*

*It was shown that the hydrophobicity in the inner side of a globular protein is very similar to the facing sides of TM regions. As can be seen in the result list, best template is an oligomer with TM regions facing each other. Considering these two statements it is not a bad prediction of reliability. If the best matching template was a lonely 2-helix TMP, that would be the indication of error. The fact, that TMFoldWeb returns the same template even at low sequence similarity (52 %) underlines its robustness and presents its independence on sequence similarity.*

*In the case of 1c17:M the current template database does not contain any structure with an average TM-score greater than 0.47. Since TMFoldRec utilizes structures solved by x-ray diffraction, the lack of the expected native structure seems reasonable and highlights the proper judgment of the algorithm.*

*Although TMFoldRec takes into account only the membrane regions of TMP sequences, the presented accuracy in fold prediction implicates that TM regions define their 3D structure themselves and their tertiary structure is not the consequence of the globular part. In this sense, modeling the membrane embedded and the soluble parts, loops (with constraints) separately is a viable solution.*

*Referees have right, there is a huge room for improvements, but a solid based step-by-step, down-to-top approximation is needed for the detailed understanding of the structure formation and stability of TMPs. In an all segment approximation there wouldn’t be any possibility to realize the remarkable contribution of TM regions in the structure.*

**Minor issues:**

**R3:** It is strongly recommended to include a flowchart diagram to describe the workflow and software implementation around the existing TMFoldRec module so that the readers can better understand what is being presented as new in this article.

***Authors’ response:****Diagram is attached as* Fig. 2*into the manuscript.*

**R3:** In the discussion section, the paragraph “if the sequence identity of the transmembrane segments is low, the relation of the two TMPs is not proved. Perhaps this lower sequence identity threshold is the consequence of the lower sequence complexity. Therefore, other information is needed to establish the relationships between TMPs, for example considering the number of transmembrane segments in addition to the sequence identity, as it was shown in the previous section.” to justify for the necessity of fold prediction appears incomplete. The purpose of fold prediction is to transfer the fold (hence function) of the hit protein to the query sequence though the sequence‑fold‑function paradigm. When sequence similarity between two homologous sequences is degenerated, having more matched fold‑critical segments (in this case TM segments) between hit and query does not automatically translate to the same protein fold. The statistical significance of the matched fold‑critical segments still requires further characterization.

***Authors’ response:****It seems our statement is not well written. Here we do not promote fold recognition, only we would like to highlight the danger of purely sequence based fold prediction and/or homology modeling, since the twilight zone of transmembrane protein is not well studied yet. In our case, the fold-critical segment is the whole membrane embedded structure. In addition, since we prefilter templates before threading based on the (predicted) topology, in such way we avoid to assign each membrane localized sequence parts to the membrane embedded structure fragments.*

*Referees have right that our method is not able to identify the native structure as a part of a bigger membrane protein. This is one of the further developmental directions of the project.*

**R3:** There exists an inconvenient issue where the FASTA sequence needs to be in caps and certain characters like ‘:’ and “(” were not allowed in the sequence descriptor. The latter is corrected for through trial and error since there are no warnings to advise the users how to correct their inputs.

***Authors’ response:****Thank you for the suggestion. The set of the enabled characters has been extended to improve usability.*

## Abbreviations

HTP: Human transmembrane proteome
PDB: Protein data bank
PDBTM: Protein data bank of transmembrane proteins
TM: Transmembrane
TMP: Transmembrane proteins
WSDL: Web service definition language
